# Transcatheter Structural Heart Interventions in the Acute Setting: An Emerging Indication

**DOI:** 10.3390/jcm13123528

**Published:** 2024-06-16

**Authors:** Nikolaos Pyrpyris, Kyriakos Dimitriadis, Panagiotis Theofilis, Panagiotis Iliakis, Eirini Beneki, Daphne Pitsiori, Panagiotis Tsioufis, Mony Shuvy, Konstantinos Aznaouridis, Konstantinos Tsioufis

**Affiliations:** 1 First Department of Cardiology, School of Medicine, National and Kapodistrian University of Athens, Hippokration General Hospital, 115 27 Athens, Greece; npyrpyris@gmail.com (N.P.); panos.theofilis@hotmail.com (P.T.); panayiotisiliakis@gmail.com (P.I.); e.beneki@hotmail.com (E.B.); daphne.pitsiori@yahoo.com (D.P.); panos.tsioufis@gmail.com (P.T.); conazna@yahoo.com (K.A.); ktsioufis@gmail.com (K.T.); 2 Jesselson Integrated Heart Centre, Shaare Zedek Medical Center, Faculty of Medicine, Hebrew University, Jerusalem 9103102, Israel; monysh@gmail.com

**Keywords:** structural heart disease, acute cardiovascular disease, cardiogenic shock, transcatheter interventions, acute myocardial infarction, transcatheter aortic valve implantation, transcatheter edge-to-edge repair

## Abstract

Structural heart disease is increasingly prevalent in the general population, especially in patients of increased age. Recent advances in transcatheter structural heart interventions have gained a significant following and are now considered a mainstay option for managing stable valvular disease. However, the concept of transcatheter interventions has also been tested in acute settings by several investigators, especially in cases where valvular disease comes as a result of acute ischemia or in the context of acute decompensated heart failure. Tested interventions include both the mitral and aortic valve, mostly evaluating mitral transcatheter edge-to-edge repair and transcatheter aortic valve implantation, respectively. This review is going to focus on the use of acute structural heart interventions in the emergent setting, and it will delineate the available data and provide a meaningful discussion on the optimal patient phenotype and future directions of the field.

## 1. Introduction

Valvular heart disease (VHD) is one of the most frequent cardiovascular pathologies, especially in the era of aging populations [1,2]. VHD can originate from a variety of pathophysiological mechanisms, ranging from anatomical (bicuspid aortic valve) to degenerative, infectious, functional, or ischemic etiologies [3]. In previous decades, the only available option for treating such pathologies was open heart surgery, but novel techniques have been developed and largely implemented into clinical practice in the last 20 years [4], which comprise the field of transcatheter structural heart interventions; these techniques have allowed interventionalists to repair or replace valvular pathologies with transcatheter procedures and with comparable short- and long-term results, compared to surgery [5,6]. This has led to the proposal of transcatheter interventions as a safe and feasible intervention in recent VHD guidelines for stable patients with a suitable phenotype and considered high-risk for surgery, for aortic and mitral disease [7], while tricuspid interventions are being recommended in expert consensus, but await more long-term data [8]. Nevertheless, a growing amount of evidence, especially in transcatheter aortic valve implantation (TAVI), is starting to support the use of such procedures in more patient phenotypes, such as those at less surgical risk and or of a younger age, thus increasing the indications for transcatheter structural interventions [9].

Cardiogenic shock (CS) is an emergent, potentially life-threatening situation, which can be complicated by VHD. Specifically, valvular emergencies can constitute up to 9% of all emergencies needing admission in a cardiovascular intensive care unit [10]. Of note, this pathology can be related to a chronic VHD, which contributes to the occurrence of left ventricular (LV) dysfunction, acute heart failure (HF), and CS; however, it can also be a result of an acute ischemic event, as in acute mitral regurgitation (MR) following papillary muscle rupture [11]. The state-of-the-art treatment of CS complicated by VHD is considered to be surgery, which directly addresses the pathology and provides immediate repair or replacement of the valve. However, currently, there are no societal guidelines specifically addressing the usability of most transcatheter interventions in the acute setting, besides recently published expert consensus [12,13]. This comes as a result of the exclusion of CS patients from landmark randomized controlled studies (RCTs) and the limited evidence of such interventions in the setting of acute HF and CS, thus leaving the decision on which intervention to perform with the heart team of each institution. Thus, in this review, we aim to provide updated, evidence-based guidance for managing VHD in the emergent setting, as well as discussing the future directions on this context.

## 2. Urgent Transcatheter Interventions for Aortic Pathologies

Aortic stenosis (AS) is the commonest VHD, with an estimated prevalence of 12.4% in the elderly, with 3.4% of the population having severe AS; rates are expected to increase in the future as a result of the aging population [14,15]. Chronic AS can be complicated by CS when specific triggers of declining cardiovascular hemodynamics impact the heart, including acute myocardial infarction (AMI), atrial fibrillation, volume overload, and sepsis. Without treatment, the survival rate of such patients is dreadful, with studies exploring the safety of balloon aortic valvuloplasty (BAV) documenting a mortality rate of up to 70% in those left without intervention, compared to those that underwent early valvuloplasty [16]. The management of such patients is challenging, as pharmacotherapy can be complex and does not always result in clinical improvement. Currently, the use of BAV is recommended by guidelines in high-risk AS patients with CS [7]. However, TAVI can be alternatively performed, instead of BAV [13], in such patients, while data show that 1.6–4.1% of the United States TAVI population have CS [17,18].

Early experience with surgical aortic valve replacement (SAVR) showed favorable results regarding mortality and symptom-free survival, with acceptable operation-related mortality rates and adverse events [19,20]. However, shortly thereafter, given the high surgical risk of these patients, transcatheter solutions started to be evaluated. BAV is a percutaneous technique, in which dilations with a balloon aim to increase the aortic valve area and reduce the transvalvular gradient. The technique was first described by Alain Cribier in 10 patients with AS and refractory to medical treatment CS, mostly as a bridge to SAVR (60%). The technique had a 20% 30-day mortality, while the remaining patients, both undergoing and not SAVR, had a significant hemodynamic improvement at repeat catheterization at approximately 6 months follow-up [21]. Several other trials were performed; however, these had increased in-hospital mortality rates following the intervention [22], reaching up to 70% in the study by Buchwald et al., with an onset of shock >48 h before the intervention being significantly related with a fatal outcome [16], which has also been confirmed by further investigations [23]. Interestingly, more recent studies report lower rates of in-hospital mortality, which have been reported as low as 33% in the largest cohort of emergent BAV [24]. These results question the effectiveness of BAV as an effective treatment for CS and AS; however, it should be noted that although they perform sub-optimally in isolation, when they are used as a “bridge therapy” to either a subsequent SAVR or TAVI, patients may have enhanced survival at 1 year [25].

Although BAV could offer a survival benefit and hemodynamic improvement in a “no-option” cohort, especially when an aortic intervention followed, the emergence of TAVI and the favorable results this technique has shown created the need for it to be tested in the emergent setting. The key studies available to date are shown in Table 1. 

Ancona et al. were one of the first group to investigate the feasibility of transapical TAVI in a previously considered contraindicated patient phenotype [26]. They included 21 patients with a diagnosis of CS, receiving a SAPIEN valve. Regarding 30-day mortality, it was significantly higher than high-risk TAVI (19% vs. 5%, *p* = 0.02), while 1-year survival was significantly lower (46% vs. 83%, *p* < 0.0001). It should be noted that all risk scores, even in comparison to a high-risk TAVI phenotype, were significantly higher in the CS group. Moreover, in the CS cohort, no technical adverse events were noted as responsible for early mortality, as all patient died from multiorgan failure. Other studies, with transfemoral TAVI and moderate number of included patients also showed a significantly increased mortality, compared with elective TAVI (33.3% vs. 7.7%, *p* < 0.0001); however, there was no difference in cumulative survival after the 30-day landmark analysis [27]. Other investigators reported lower mortality rates (11.8%) and severe paravalvular leaking (5%), with an acceptable occurrence of stroke (2.0%) and vascular complications (5.9%), but increased rates of acute kidney injury (AKI) (34%) [28], while Landes et al. showed comparable mortality and no difference in periprocedural complications of urgent TAVI with elective TAVI, in patients with acute HF [29].

Kolte et al. reported a larger analysis on this context, investigating urgent and emergent TAVI in the Society of Thoracic Surgeons/American College of Cardiology Transcatheter Valve Therapy (STS/ACC TVT) registry between 2011–2017 [17]. Of the 40,042 patients, 9.9% had an emergent or urgent procedure, with a lower device success rate compared to elective procedures (92.6% vs. 93.7%; *p* = 0.007), but no meaningful clinical differences in comparison to elective TAVI, as adverse events were similar between groups, with the exception of AKI and/or new dialysis (8.2% vs. 4.2%; *p* < 0.001). Regarding mortality, urgent/emergent TAVI was significantly related with excess 30-day [8.7% vs. 4.3%, adjusted hazard ratio (HR): 1.28, 95% confidence interval (CI): 1.10 to 1.48] and 1-year events (29.1% vs. 17.5%, adjusted HR: 1.20, 95%CI: 1.10 to 1.31). However, the authors highlight the acceptable outcomes of urgent/emergent TAVI, especially in the setting of CS.

A following analysis of subsequent data from the STS/ACC TVT Registry [30], including patients with CS undergoing TAVI compared to high-risk TAVI patients, also showed an increased 30-day mortality (19.1% vs. 4.9%), which remains elevated for at least 1 year after the procedure, proportionally to the degree of shock. However, in contrast to previous data, they showed an increase in 30-day reinterventions (3.7% vs. 0.7%, *p* < 0.001) and unplanned cardiac surgery (6.8% vs. 1.3%, *p* < 0.001), along with AKI. Independently associated with 30-day mortality were cardiac arrest and the need for pre-procedural mechanical circulatory support (MCS), especially bypass.

Finally, the most recent analysis of the STS/ACC TVT registry aimed to provide an update to several questions regarding the safety and feasibility of urgent TAVI [18]. Of the total registry, 1.6% of patients presented with CS. Successful implantation was achieved in 97.9% of patients, with technical success in 94.5%. In a propensity-matched cohort, CS was associated with increased mortality in all in-hospital (9.9% vs. 2.7%), 30-day (12.9% vs. 4.9%), and 1-year (29.7% vs. 22.6%) follow-ups. However, in the landmark analysis after 30 days, the risk of mortality at 1 year was similar (HR 1.07, 95% CI 0.95–1.21). Regarding quality of life, those alive at 1 year reported a significant improvement. Finally, predictors of 1-year mortality included older age, peripheral artery disease, dialysis, presence of an implantable cardioverter defibrillator, New York Heart Association (NYHA) class III/IV, and being immunocompromised. 

Steffen et al. [31] aimed to evaluate patients with acute heart failure, either fulfilling the criteria for CS or not, in comparison with patients undergoing elective TAVI. A total of 2930 patients were enrolled, of whom 47 had CS and 132 had severe decompensation without CS, while the remaining were elective procedures. In comparison to the decompensated patients, CS patients had significantly higher STS scores and were more frequently male. Regarding 90-day mortality, CS patients had the most increased rates (42.6%), as compared to non-CS (15.9%) and elective patients (5.3%) (*p* < 0.01). However, after the landmark 90-day analysis and for two years follow-up, there was no significant difference in mortality between groups. Finally, predictors of 90-day mortality were mechanical ventilation, hemofiltration, elevated C-reactive protein, elevated bilirubin, and hypotension before the procedure. 

Similarly, Castello et al. [32] showed that in the unadjusted population, urgent TAVI was associated with an increased mortality rate (25.3 vs. 15.1%, *p* = 0.043), 30-day cardiovascular mortality (17.5 vs. 4%, *p* = 0.001), major bleeding (11.5 vs. 4.1%, *p* = 0.018), and vascular complications (11.5 vs. 4.6%, *p* = 0.031), as well as increased hospital stay, compared to elective TAVI. However, after adjustment for baseline characteristics, the only outcome that remained significantly different was the length of hospital stay.

Recently, Bandyopadhya et al. aimed to directly compare urgent BAV and TAVI in the acute setting [33]. In a propensity-matched analysis, the investigators reported a non-significantly different mortality rate between the two procedures (HR 0.79; 95%CI 0.51–1.21; *p* = 0.29), with increased post-procedural AMI in the BAV groups and increased permanent pacemaker, major bleeding, and complete heart block events in the TAVI group. Although no significance was found in AKI events, and these were lower in the TAVI arm. These findings are in concordance with a previous smaller study, which showed non-significant numerically lower mortality rates, in both immediate procedural (8.7% vs. 20.3%; *p* = 0.19) and 30-days follow-up (23.8% vs. 33.0%, *p* = 0.40), with the use of TAVI; however, TAVI was associated with significantly increased vascular complications [24].

Given the lack of randomized data, a meta-analysis by Wernly et al. showed that in patients with CS, TAVI is associated with a 30-day mortality of 22.6%, major bleeding events of 5.8%, and stroke events of 4% [34]. In contrast, BAV was associated with a 30-day mortality rate of 46.2%, major bleeding rate of 10%, and stroke rate of 0.7%. Thus, as TAVR has a survival benefit, is associated with immediate, long-term hemodynamic improvement and has no need for staged operations, it could be considered the “go-to” choice, leaving BAV as a bridge therapy in centers with no TAVI experience. Finally, regarding adverse events and safety, a more recent meta-analysis comparing urgent with elective TAVI and including 84,495 patients reported an increased 30-day mortality (RR: 2.53, 95%CI: 1.81–3.54), in-hospital mortality (RR: 2.67, 95%CI: 1.94–3.68), periprocedural vascular complications (RR: 1.91, 95%CI: 1.28–2.85), and AKI risk (RR: 2.83, 95% CI: 1.93–4.14), with no other differences regarding outcomes [35].

Finally, urgent TAVI has been reported in patients with cardiogenic shock requiring mechanical circulatory support (MCS), thus showcasing the potential feasibility of the procedure in hemodynamically unstable patients. Several case reports have been published. In more detail, Osawa et al. [36] reported the case of 64-year-old male with HF due to severe AS due to a bicuspid aortic valve, further complicated by cardiogenic shock. The patient was placed into veno-arterial extracorporeal membrane oxygenation (ECMO), while an intra-aortic balloon pump was also placed in order to be transferred to a reference medical center. On day two after presentation, the investigators decided to perform urgent TAVI, which improved the hemodynamic parameters and led to the discharge of the patient after 27 days. Similarly, El Tahlawy et al. [37] report the case of a 57-year-old male, presenting with AMI and critical AS. The team originally performed BAV under ECMO; however, afterwards they completed a TAVI procedure, in order to account for the regurgitation caused by BAV. Although Impella could be considered not the optimal option for aortic valvulopathies, experience with BAV has shown that Impella implantation was successful in all instances after emergent BAV [38]. In patients undergoing TAVI, Impella as a temporizing measure prior to TAVI has also been described [39]. In an analysis by Almajed et al., regarding emergent TAVI and BAV in a population consisting of 120 patients (26 TAVI, 94 BAV, 53.9% CS), in specific for etiology analysis, Impella-TAVI in CS patients had a 30-day mortality rate of 34.6%, while Impella-BAV had a 30-day mortality rate of 44.2%. In these cases, Impella was used prior to the procedure in order to achieve hemodynamic stability [40]. Similar high mortality rates (40%) in patients requiring Impella have been reported by Almalla et al. [41], with low device-associated complications.

The aforementioned results showcase the safety and feasibility of urgent TAVI in the context of CS, while its performance is shown to be better than the current alternative, i.e., BAV. Moreover, it should be highlighted that despite some studies and meta-analyses showing increased periprocedural complications rates in the urgent procedures, especially regarding renal events (AKI), most large registry data show no difference in mortality, especially in the long term, and no significant periprocedural adverse events, besides renal failure. This showcases the safety and effectiveness of TAVI in an urgent setting. However, it has to be recognized that the studies comparing the two interventions head-to-head are limited, as are the randomized data in this topic. Therefore, more RCTs comparing the two interventions are needed, in order to validate the superiority of TAVI compared to surgery and BAV and to lead to recommendations concerning TAVI in this setting. Furthermore, there might have been an allocation bias in the aforementioned trials, as patients offered to undergo surgery may have been those that the heart team considered would have the best survival odds. This should be explored in more depth in future RCTs. Finally, some technical considerations still have to be answered. The implantation of a prosthetic valve through TAVI, contrary to BAV, aims not only to offer a bridge to surgery, but also a permanent solution (destination therapy). Considering this, further investigations should explore which valve type is superior in CS patients, as such patients, at the time of implantation, have altered hemodynamics which could impact short-term valve function. Furthermore, despite the urgency, proper planning must be undertaken, especially in patients with a life expectancy that could require a valve re-intervention. It is key to identify which imaging modality can provide the most information in the least amount of time, in order to proceed with the intervention. Moreover, access to coronaries and position of the valve, as well as differences between valvular devices that could facilitate such aspects, is necessary when planning an urgent TAVI, in order to provide optimal care. Such investigations should be pursued in the future, in order to provide more answers regarding procedural optimization. Finally, as the use of MCS is undertaken with limited evidence and no large body of data, future studies should further investigate the optimal timing and MCS modality in this distinct, high-risk category of CS patients requiring mechanical support.

## 3. Urgent Transcatheter Interventions for Mitral Pathologies

### 3.1. Mitral Regurgitation

Acute mitral regurgitation (MR) is a complex medical pathology, with its etiologies ranging from acute MR due to AMI, exacerbation of a previously existent MR in the presence of a triggering factor, or acute chord rupture [11]. Acute MR, especially secondary to ischemia, even though being less prevalent in the era of primary percutaneous coronary interventions (PCI), is known to be associated with increased mortality rates, which can exceed 50% in one-year follow-ups [42]. Significant MR in the setting of AMI can be a result of direct damage to the valvular mechanism (chordae rupture, primary MR) or a result of immediate LV remodeling due to ischemia (secondary MR). In series of PCI patients, it has been found that at the time of primary PCI, approximately one-third of patients have any type of MR, while 10% have severe MR. However, in two-thirds of patients, the pathology will improve or not change in severity, while in the remaining one-third it will worsen [43]. In a similar manner, the presence of chronic MR in patients with acute decompensated HF and CS further contributes to the pathophysiology of the syndrome, compromising patient’s hemodynamics and increasing adverse outcomes. 

Therapeutically, these patients have limited options, with the main agents being inotropes and vasoconstrictors, with escalation to surgical treatment in refractory CS not responding to optimal medical treatment. However, given the high 30-day mortality risk of surgical interventions in such high-risk patients, which can reach up to 22.5% in overall acute MR and even higher in those with an ischemic substrate [44], the option of transcatheter interventions, and particularly transcatheter edge-to-edge repair (TEER) has recently become appealing. Thus, a number of studies have evaluated the role of urgent TEER in this setting (Table 2).

The first data come from case reports [55,56] and small case series [45]. In particular, Adamo et al. [45] present five patients with AMI, CS (80%) or pulmonary edema (20%) and concomitant severe MR, who underwent a MitraClip procedure. The patients were dependent on MCS and intravenous inotropes. The procedure was performed at a mean time of 53 days from the index event, with procedural success (MR < 2+) achieved in all patients. Furthermore, hemodynamics and pulmonary pressure were substantially improved. All patients were weaned from MCS and intravenous therapy. Four out of five patients were alive at 30-day follow-up, with the one mortality event not being related to cardiovascular pathologies.

Later studies employing a larger number of patients further confirmed the previous findings. Haberman et al. presented one of the first large series of patients receiving a MitraClip device, as a salvage procedure, in the context of AMI and secondary MR [46]. The investigators included 20 patients with a TEER operation in the 90 days following an AMI, due to the failure of medication to improve patient status. All patients had at least severe (3+) MR, while 40% of patients had CS. The procedure was performed at a mean of 30 days (range 7–90) after the AMI event, with more severe patients (mechanical circulatory support or ventilation) receiving the operation earlier. Procedural success was 95%, while a significant reduction in MR was achieved in the majority of patients (MR1+ in 12; MR2+ in 7 patients). Significant hemodynamic improvement was also noted, albeit without a significant change in LVEF. Finally, these results were sustained at a median follow-up of 15 months. 

In concordance are the results that have been reported in a prospective trial by Esteves-Loureiro et al. [47], where MitraClip surgery in 44 patients with AMI and severe MR resulted in high technical success (86.6%), with a sustained reduction in MR grade and NYHA class improvement observed at 6 months follow-up.

Recently, Haberman et al. presented the results of the IREMMI registry, which consisted of patients with severe MR and AMI, treated either with medical therapy, surgical valve replacement or repair or TEER [48]. The study comprised 471 patients, namely 205 undergoing any intervention (106 undergoing surgery, 99 TEER) and 266 being conservatively treated. Of note, patients assigned to interventions were of worse clinical status at baseline, with a Killip class greater than 3 being significantly more frequent in the interventions arm (60% vs. 43%; *p* < 0.01). However, this did not translate into clinical compromise, as they had lower in-hospital (11% vs. 27%; *p* < 0.01) and 1-year mortality (16% vs. 35%, *p* < 0.01), compared to conservative treatment. Regarding the comparison between the two interventions, procedural success was similar between surgery and TEER; however, there was a significant benefit of TEER in both in-hospital (6% vs. 16%; *p* = 0.03) and 1-year (17% vs. 31%; *p* = 0.04) mortality rates. Interestingly, a sub-analysis of the study for patients in CS showed no difference in TEER safety, efficiency, and post-procedural adverse events between CS and non-CS groups, under the condition that hemodynamic stabilization has been achieved prior to TEER [57].

Evolving from studies primarily assessing the setting of AMI, Simard et al. analyzed the role of urgent TEER in patients presenting with CS, from the STS/ACC TVT registry [49]. This study enrolled 3797 patients, of which 16.5% had ACS. Device success, defined as reduction in MR by ≥1 grade and MR grade ≤ 2+, was achieved in 85.6% of patients. In-hospital mortality was significantly increased in the failure arm (16.4% vs. 9.1%; *p* < 0.001). In a comparison of device success versus failure at 1 year, device success was associated with significantly lower all-cause mortality (34.6% vs. 55.5%; adjusted HR: 0.49; 95%CI: 0.41–0.59), as well as a composite of mortality and HF hospitalizations (29.6% vs. 45.2%; adjusted HR: 0.51; 95% CI: 0.42–0.62). In concordance, the analysis of the MITRA-SHOCK trial [50], also enrolling patients with CS and severe MR, showed that a successful intervention is an independent prognostic factor of both 30-day (HR = 0.12; 95%CI: 0.03–0.55) and 6-month survival (HR = 0.22; 95%CI: 0.06–0.84).

Cheng et al. [51], assessing 29 patients with severe MR requiring continuous inotrope support or MCS, showed that the procedure has similar effectiveness in MR reduction and intraprocedural complications with elective TEER, while the majority of patients will survive both 30-day (82.8%) and 6-months follow-up (75.6%), following successful down-titration of inotropes and a transition to only pharmacotherapy, heart transplantation, or durable MCS.

Similar results, with significantly decreased 30-day and 1-year mortality rates, have been also found by Tang et al. in a US nationwide analysis [52], while the 30-days mortality rate was lower than that predicted by the STS score. It should be noted that the investigators found a significant, approximately 3-fold, increase in the use of mitral TEER in CS. However, the benefit of MitraClip in this study, despite being consistent among most subgroups, was observed only in patients not requiring MCS or hemodialysis (*p* for interaction = 0.004 and 0.011, respectively). 

Finally, some investigators examined the efficacy of urgent TEER papillary muscle rupture. Haberman et al., analyzing data from the IREMMI study, evaluated 23 patients with papillary muscle rupture (87% in CS). TEER was performed in a median of 6 days post-AMI, with a procedural success rate of 87% and significant MR reductions (57% MR grade 0–1+) [53]. Similar success has been also shown by other investigators [54]. 

It is of note that, similarly to AS, significant experience specifically addressing the need and outcomes in patients requiring MCS is not available, despite a lot of patients in large studies having required MCS. Several case reports have reported the safety and efficacy of using both Impella [58,59,60] and ECMO [61]. Interestingly, the study by Simard et al. [49] did not find any difference in device success between different CS patient phenotypes, including those requiring MCS. Finally, Vandenbriele et al. [62], reporting six patients with CS and acute severe MR requiring Impella as bridge to the procedure, and showed that combined Impella-MitraClip was feasible; after TEER, it was possible to wean the patients off MCS. The survival rate to discharge was 86%. Given the positive results of MCS use in CS [63], it is necessary to study the use of MCS in urgent TEER and its impact on outcomes in future trials.

The aforementioned results for the role of TEER in the urgent setting have been also confirmed by recent meta-analyses, providing additional promising evidence on the selection of urgent TEER, contrary to surgery, in the acute setting [64,65]. It is noteworthy that the device success and complications rates were relatively high and low, respectively, especially considering elective procedures’ rates. However, it should be recognized that there are some limitations to the above studies. The results cannot be generalized for mitral TEER in general, as the vast majority of studies used only one of the available devices (MitraClip). Experience with other systems is necessary, in order to generalize these findings. Furthermore, the inclusion criteria of each study, and particularly in regard to the definition of CS, were largely different. Future efforts should include uniform definitions of CS and severity assessment, such as the Society for Cardiovascular Angiography and Interventions (SCAI) shock grades [66]. As these studies were executed at different centers, with varying levels of expertise in urgent and elective TEER, operator-dependent differences in procedural planning and shock management may have influenced these results. Finally, the observational nature of these trials may introduce a selection bias, as patients that were more probable to survive may have been offered the intervention. Such limitations have to be addressed, and, therefore, the results of the first RCT in this context, i.e., CAPITAL MINOS, are largely awaited [67].

Despite the positive results of the up-to-date experience with TEER in CS, in both AMI and non-AMI patients, there are some technical questions inherent to the procedure that remain to be answered. Firstly, the long-term safety and efficacy of TEER remains not fully understood, as only a few trials have reported extensive long-term results. Furthermore, TEER-induced fibrosis in the mitral valve may limit subsequent surgical options, especially surgical repair, which may be considered in chordal rupture [68]. Thus, in such patients, TEER could complicate later surgical interventions and, despite showing positive outcomes, it may be used only as a salvage in prohibitive surgical risk patients. Moreover, the anatomy of the valve has to be considered thoroughly before deciding to perform the intervention, as the concept “one size fits all” does not apply in the setting of MR. Finally, the immediate resolution of MR may not always result in a clinical benefit. In particular, the underrecognized afterload mismatch, which is defined as the acute impairment of LV systolic function as a consequence of increased afterload after correction of the regurgitation, has been described in the literature after TEER and is linked to worse outcomes [69,70]. This could be a dreadful complication in an already deteriorating LV, and although there is no clear evidence of LV ejection fraction drop in the aforementioned studies, it should be more extensively studied, especially as in the setting of CS, the medical therapy may have masked this observation. 

### 3.2. Mitral Stenosis

As with all other valvulopathies, severe mitral stenosis (MS), mostly secondary to rheumatic heart disease, can also be related to CS. However, this presentation is infrequent and is vaguely described in the current literature, with only case reports exploring this topic. Regarding its medical management, the typical management of CS and other related comorbidities must be undertaken; however, when the culprit for CS is considered to be the MS, it should be efficiently addressed during patient presentation.

The percutaneous mitral balloon valvuloplasty (MBV) has been one of the cornerstones for managing severe MS, with it being recommended in the most recent European guidelines for managing VHD. Given its less invasive and less risky profile compared to open surgery, MBV could be an interesting option in patients with severe MS and CS or acute decompensated HF. Mostly case reports and small studies to date have evaluated the safety and feasibility of this hypothesis, where MBV has been shown to be safe and feasible in the acute management of CS, providing immediate hemodynamic and symptomatic improvement [71,72,73,74], even in patients during pregnancy [75]. More recently, given the emergence of transcatheter-delivered prosthetic mitral valves, the role of TMVR in the emergent setting seems promising as an alternative to surgery. However, there is a lack of evidence in this context and future investigations should be undertaken.

## 4. Other VHDs: Is There a Need?

Along with the most commonly investigated VHD and CS presentations in clinical studies, it is well established that all VHD, under the appropriate triggers, can initiate a vicious cycle of hemodynamic instability, leading to CS. Among the less evaluated pathologies in CS are aortic regurgitation (AR) and tricuspid regurgitation (TR). These pathologies are, potentially, less studied in this context, as it is more uncommon, especially for right-heart valvulopathies, to be associated with CS, although advanced right HF can also result in severe shock due to decreased forward cardiac output [76]. In the literature, AR has mostly been examined in relation to transcatheter interventions in CS, while there are no data regarding tricuspid valvulopathies.

In cases of AR-complicated CS, medical management is key in order to hemodynamically support the patient, while atrial pacing may be needed in order to decrease the diastole time [77]. Surgery has a key place in improving these patients; however, similar to other valvulopathies, given the high-risk of such groups, it can be associated with high mortality (although robust data are lacking). The role of TAVI in this context is not extensively explored. To date, several case reports [78,79,80] have demonstrated the feasibility of urgent TAVI for AR. Furthermore, a small cohort study by Achkouty et al. enrolled five patients with AR and CS, in whom a TAVI was implanted, as they were of prohibitive risk for surgical intervention [81]. The success rate of the procedure was 100%, with no device migration or procedural adverse events. Post-procedurally, mild AR was noted in two patients. At 30 days follow-up, four patients were alive with an uncomplicated recovery, while there was one death recorded due to pulmonary sepsis and multiorgan dysfunction, without indications of prosthesis endocarditis. The lack of data highlights the need for more studies, with an increased number of patients and extended follow-up, in order to make any recommendations. However, as TAVI is still a new frontier in the management of stable AR, with no guideline recommendations regarding its use and mostly recently published, short-term results [82], much research is needed in order to better understand the safety and efficacy of TAVI in stable AR, at first, and then in AR-related CS. Finally, it should be considered that TAVI has a contraindication in endocarditis and aortic dissection, which comprise the two most common etiologies of acute AR, thus potentially limiting the wide application of the intervention in these underlying AR pathologies.

Transcatheter interventions have been considered in cases of degenerated prosthetic valves, with particularly limited experience and in patients of prohibitive surgical risk. In cases of degenerated aortic prostheses and an emergent setting, there have been several case reports on patients with endocarditis [83,84], early failure of the prosthetic valve [85], and acute decompensated HF or CS secondary to structural valve dysfunction [86,87,88,89]. Although in these instances the feasibility of TAVI has been proven, no case series or observational study has thoroughly evaluated this potential TAVI indication. Similarly, in bioprosthetic mitral valve stenosis, several case reports of mitral valve-in-valve implantation in patients with CS or not fulfilling CS criteria but with hemodynamic instability have been presented, with positive results [90,91,92]. Moreover, a study by Elmously et al. [93], consisting of a majority of CS patients (63%) with bioprosthetic mitral valve failure, showed that transapical transcatheter valve-in-valve implantation had 100% technical success, with significant reductions in the transvalvular gradient (12 ± 5 mmHg to 5 ± 3 mmHg; *p* = 0.0005). At the time of the follow-up (mean 339 days), 15.8% of patients had developed a trace transvalvular regurgitation, while approximately 90% of patients were at NYHA class I or II. It is important to highlight that these early results have to be confirmed by further studies and analyses. Thus, currently, appropriate medical management, MCS, and surgery for such cases remains essential, until further data become available.

## 5. Future Directions

As previously discussed, transcatheter structural interventions in the acute setting are a novel frontier in both clinical practice and research, aiming to provide a safe and effective option in those that are at high-risk for surgery, or even to ultimately replace surgical interventions as a lower-risk and more time-effective alternative (Graphical Abstract). It is evident that in some valvulopathies (AS, MR), there is extensive evidence on the safety and efficacy of the respective procedures, while in others experience is limited to case reports and series. However, currently, only TAVI in AS is recommended by societal expert consensus in the acute setting, while in the absence of randomized data other interventions, such as TEER, are to be considered only in patients of prohibitive surgical risk [13]. Along with the much-awaited RCTs, especially in MR and TEER, which is the most quickly evolving indication, the optimal timing of intervention needs to be identified. Specifically, the current indication for surgery in patients with VHD and CS consists of the failure of conservative management and MCS to stabilize the patient or deterioration of the patient. However, there is no consensus regarding what the most appropriate time is, i.e., which laboratory, imaging, and interventional values should be the cut-off, in order to decide to intervene. It is of utmost important is to decide in which patients it would be futile to proceed with an intervention, such as patients with multiorgan dysfunction. Furthermore, assessing the patient’s hemodynamic status by invasive hemodynamics could discriminate patients in CS where an urgent procedure, contrary to an elective, should be performed. It is vital, therefore, to investigate in future trials which markers and what thresholds should be used in decision-making.

Of note, in the presence of multiple combined valvulopathies complicating CS, it is currently unknown and unexplored which intervention should be performed first. In the era of surgical interventions, and in cases where the patient is of acceptable surgical risk, it should be logical to pursue a “one-stop” surgery for both pathologies. However, in patients pursuing a transcatheter approach, there is less clarity about which intervention should come first. Algorithms for stable patients indicate that, in the presence of severe AS and significant/severe MR, TAVI should be performed, and MR should be medically treated and followed up. However, in cases of severe MR and moderate AS, if MR is the prognostically leading pathology, mitral intervention should be performed, followed by AS follow-up [94]. In the urgent setting, given the lack of evidence, no such recommendations exist in consensus documents or guidelines. However, it should be highlighted that the heart team should decide to treat the culprit of CS, taking into consideration presentation, prior medical history, prior echocardiography, and the mechanism of valvular heart pathology. Future trials should, thus, address the timing of interventions in combined VHDs. Finally, the hypothesis of concomitant TAVI and TEER during the same admission has been described in stable patients, showing no difference in in-hospital mortality, compared to staged procedures; however, increased rates of acute kidney injury, vascular complications, need for percutaneous coronary intervention, mechanical support, and pacemaker implantation are observed [95]. Interestingly, other investigators also found increased mortality, compared with isolated TAVI [96] and TAVI followed by TEER [97], which could make this approach unappealing for CS patients. 

Furthermore, in the context of acute interventions, when using transcatheter procedures, it should be decided whether they should serve as bridge, like BAV, or as a destination therapy. As aforementioned for MR, specific MR phenotypes, which require surgical replacement, cannot be as easily performed with a bridge TEER as without. On the other hand, a consequential benefit of transcatheter valve replacement or repair is the opportunity to offer immediate treatment, as opposed to other techniques, like BAV. Thus far, although none of the studies have evaluated long-term results, studies reporting 1-year outcomes show positive results, with similar to elective TAVI mortality rates after the initial critical period [18], thus signaling the safety of the described procedures as a destination treatment. Although more extensive follow-up is needed in order to evaluate long-term durability, similarly to non-urgent procedures, the benefit of the immediate resolution of the pathology is one of the key points of urgent transcatheter interventions, that ultimately supports its role in this context.

As described, patients with CS could have both a valvular pathology and a concomitant AMI. In such instances, revascularization of the culprit is of outmost significance, as some valvulopathies (especially in the setting of MR) can be reversed in severity after revascularization [43]. However, the question arises whether a concomitant PCI and transcatheter heart intervention could be performed in these patients. Concomitant PCI has been described only in addition to TAVI, with comparable results to a stepwise strategy [98,99]. Even in stable patients, the timing of PCI is controversial, with a strategy of pre-intervention PCI being most recommended [100]. A combined strategy could offer a lower cost and fewer vascular complications, given that the same access can be used for both interventions. However, especially in the context of CS and associated tissue hypoperfusion, the combined contrast of PCI and contrast-requiring structural heart intervention (i.e., TAVI) could increase the risk of severe AKI or contribute to the deterioration of an already compromised renal function, especially as the CS-TAVI population is already at an increased risk for AKI, as mentioned. Employing techniques of ultra-low or zero contrast PCI [101], especially mediated by intravascular imaging [102], which have been also tested in the acute setting, could facilitate a combined intervention and reduced associated risks. However, in a cohort of such high-risk individuals, robust data on the benefit of a combination, rather than a staged-up procedure, need to be available in order for this hypothesis to enter clinical practice.

Finally, it is evident that urgent transcatheter structural interventions have to be performed at experienced, high-volume reference centers, with extensive experience in the procedures in stable patients as well. Organizing such centers would be significant in the optimal management of CS patients. Moreover, the need for cooperation between cardiovascular professionals needs to be highlighted, as in the setting of a transcatheter intervention in a CS patient, a handful of experts should be directly involved in the care of the patients, including acute cardiovascular, advanced HF, cardiovascular imaging, intensivist, interventional cardiology, and cardiac surgery physicians [103]. The design and implementation into clinical practice of shock teams is, therefore, advisable, as it could help decide the optimal plan of treatment and to discuss potential interventions, which could be particularly helpful as currently there are no guideline-endorsed directions on such interventions. A heart-team approach should, therefore, be initiated in designated shock centers, where all available treatment options should be available and initiated when deemed appropriate.

## 6. Conclusions

Valvular heart disease in the acute patient is a promising novel indication for transcatheter structural heart interventions, with most data documenting its safety and efficacy primarily in MR and AS. Further investigations, consisting of RCTs, as well as including other valvulopathies, are needed in order to include such interventions in clinical practice, while finding the optimal timing and patient phenotype to benefit from such interventions is essential in order to provide optimal care.

## Figures and Tables

**Table 1:** Key studies evaluating TAVI in the acute setting.

Study	Year	Study Design	Patient Characteristics (n)	Mean STS and EuroScore of Patients in CS		Outcomes	
				STS Score	EuroScore	Mortality	Adverse Events
Ancona et al. [26]	2012	Observational	21 urgent TAVI patients, with pre-operative CS	50.8 ± 28.1%	73.1 ± 18.9%	30 days: CS 19%, non-CS 5%; *p* = 0.02 1 year: CS 54%, non-CS 17%; *p* < 0.0001	NR
Frerker et al. [27]	2016	Observational	27 urgent TAVI patients due to CS	NR	60.4 ± 21.1%	30 days: CS:33.3%, non-CS: 7.7%; *p* < 0.0001 1 year: CS 40.7%, non-CS 17.3%; *p* = 0.0009 No difference in 30-day landmark analysis	CS was associated with increased AKI rates (29.6% vs. 5.0%; *p* = 0.0001) No significant differences in other events
Fraccaro et al. [28]	2020	Observational	51 urgent TAVI patients due to CS	19 ± 15%	NR	30-day: 11.8% 1-year: 25.7%	Stroke: 2.0% Vascular complications: 5.9% AKI: 34% Moderate/severe PVL: 5%
Landes et al. [29]	2016	Observational	27 urgent TAVI patients due to CS, compared with elective TAVI (158)	9.7 ± 6.1	10 ± 8.4	30 days: similar mortality rates of elective (3.8%), semi-elective (4.3%), and urgent (3.7%)	No difference in adverse events between the procedures
Kolte et al. [17]	2018	Observational	3952 urgent TAVI patients, 36,090 elective TAVI	Urgent TAVI: 11.8 (7.6–17.9); Elective TAVI: 6.1 (4.1–9.1)	NR	Significantly increased 30-day (8.7% vs. 4.3%) and 1-year (29.1% vs. 17.5%) mortality in the urgent TAVI population	Increased rates of AKI or new dialysis (8.2% vs. 4.2%; *p* < 0.001)
Masha et al. [30]	2020	Observational	2220 TAVI patients with CS vs. 12,851 high-risk patients	9.8 (CS) vs. 10.3 (non-CS)	NR	30-day: 19.1% vs. 4.9%	Significantly increased rates of 30-day stroke (4.0 vs. 2.6%), new hemodialysis (4.0% vs. 1.6%), reintervention (3.7% vs. 0.7%), and unplanned cardiac surgery (6.8% vs. 1.3%)
Goel et al. [18]	2023	Observational	5006 urgent TAVI patients with CS, 304,499 elective TAVI (4952 matched)	CS: 10.76 ± 10.362; non-CS: 4.88 ± 3.993	NR	Increased in-hospital (9.9% vs. 2.7%), 30-day (12.9% vs. 4.9%), and 1-year (29.7% vs. 22.6%) mortality, compared to elective TAVI In the landmark post-30-day analysis, similar 1-year mortality (HR 1.07)	Increased rates of in-hospital stroke (2.89% vs. 1.45%; *p* < 0.0001), new dialysis (3.53% vs. 1.11%; *p* < 0.0001), major vascular complications (2.32% vs. 1.31%; *p* = 0.0002), life-threatening bleeding (2.46% vs. 0.65%; *p* < 0.0001), and PCI (1.51% vs. 0.67%; *p* < 0.0001) in comparison to elective TAVI Similar 1-year results
Steffen et al. [31]	2022	Observational	47 CS-related urgent TAVI vs. 132 non-CS-related urgent TAVI Control with 2745 elective procedures	CS: 15.6 (8.0–32.1) Non-CS: 5.5 (3.9–8.5)	NR	90-day: CS: 42.6%; urgent non-CS: 15.9%; elective: 5.3% (*p* < 0.01) No difference at landmark analysis post-90 days	Increased new pacemaker implantation in the non-CS arm (22.7% vs. 8.5%; *p* = 0.03)
Castello et al. [32]	2023	Observational	79 urgent TAVI vs. 219 elective TAVI	Urgent: 7.09%; Elective: 4.4%	Urgent: 9.26%; Elective: 5.17%	30-day: urgent: 25.3; elective: 15.1%; *p* = 0.043 30-day CV: 17.5 vs. 4%, *p* = 0.001	Significantly increased rates of life-threatening bleeding (11.5 vs. 4.1%; *p* = 0.018), vascular complications (11.5 vs. 4.6%; *p* = 0.031), and hospital stay (28 vs. 12 days, *p* = 0.0001) in the urgent TAVI No differences (besides hospital stay) after adjustment for baseline
Bongiovanni et al. [24]	2018	Observational	23 urgent TAVI patients vs. 118 urgent BAV patients	NR	TAVI: 37.7% ± 18.1; BAV: 35.3% ± 20.8	30-day CV: TAVI: 23.8%; BAV: 33.0%; *p* = 0.40	Major vascular complications (17.4% vs. 3.4%) and stroke (8.7% vs. 0%) were significantly more frequent after urgent TAVI
Bandyopadhya et al. [33]	2020	Observational	2136 urgent TAVI patients vs. 1328 urgent BAV patients	NR	NR	After propensity matching, no difference in 30-day mortality (HR: 0.79; 95%CI: 0.51–1.21; *p* = 0.29)	Lower odds of developing AMI in TAVI (OR:0.56; 95%CI: 0.34–0.92; *p* = 0.02) Higher risk of pacemaker implantation (OR: 17.00; 95%CI: 9.13–31.97; *p* < 0.001), major bleeding(OR: 1.93; 95%CI: 1.60–2.30; *p* < 0.001), and complete heart block (OR: 8.40; 95%CI; 5.10–14.01; *p* < 0.001) with urgent TAVI

Abbreviations: CS: cardiogenic shock; TAVI: transcatheter aortic valve implantation; BAV: balloon aortic valvuloplasty; AKI: acute kidney injury; CV: cardiovascular; STS: Society of Thoracic Surgeons; HR: hazard ratio; OR: odds ratio; CI: confidence interval; NR: not reported.

**Table 2:** Key studies evaluating mitral TEER in the acute setting.

Study	Year	Study Design	Patients (n)	Setting	Outcomes		
					Mortality	MR Reduction	Functional Outcomes
Adamo et al. [45]	2017	Observational	5 patients undergoing urgent TEER	100% AMI	1 patient died at 47 days (not CV-related)	MR ≤ 2+ in all patients post-procedurally At 30-day follow-up, 1 patient had MR recurrence (3+)	NR
Haberman et al. [46]	2019	Observational	8 patients undergoing urgent TEER	100% AMI	1 in-hospital death 1 mortality event at follow-up (unknown etiology)	Post-procedural MR 1+ in 12 patients and 2+ in 7 Non-significant changes at median 15 months follow-up	NYHA class I/II in 17 patients at follow-up (*p* < 0.01)
Esteves-Loureiro et al. [47]	2020	Observational	44 patients undergoing urgent TEER	100% AMI	30-day: 9.1% 6 months: 18.2%	MR ≤ 2 at 6 months in 72.5% of patients (vs. 0% at baseline, *p* < 0.01)	75.9% were NYHA class I/II at 6 months (vs. 15.9% baseline, *p* < 0.01)
Haberman et al. (IREMMI Registry) [48]	2022	Observational	471 patients: 205 intervention (106 surgery, 99 TEER) and 266 conservative	100% AMI	In hospital mortality: intervention vs. conservative: 11% vs. 27%; *p* < 0.01 and TEER vs. surgery: 6% vs. 16%; *p* = 0.03 1-year mortality: intervention vs. conservative: 16% vs. 35%; *p* < 0.01 and TEER vs. surgery: 17% vs. 31%; *p* = 0.04	Significant and sustained reductions in MR in intervention arm at 1 year (MR ≤ 2 in TEER 85%; surgery: 90%)	Significant and sustained NYHA class I/II at 1 year in intervention arm (TEER 61%; surgery: 57%)
Simard et al. [49]	2022	Observational	3797 patients undergoing urgent TEER	Patients with CS (16.5% ACS), comparison of device success and failure arms	Lower all-cause mortality in the device success group at 1 year (34.6% vs. 55.5; *p* < 0.001)	Post-procedurally, MR grade ≤ 2+ was achieved at 88.2% and MR reduction of at least 1 grade at 91.4%	NR
Falasconi et al. [50]	2021	Observational	31 patients undergoing urgent TEER	Patients with CS (54.8% ACS)	30-day: 22.6% 6-month 54.8%	Post-procedural MR ≤ 2 in 27 patients Discharge MR ≤ 2 in 18 patients	NR
Cheng et al. [51]	2019	Observational	29 patients undergoing urgent TEER	Patients with refractory CS, in need of MCS or continuous inotropes (ischemic CS 34.5%)	Hospital discharge: 17.2% 6-month: 24.4%	Post-procedurally, 89.7% of patients moderate or less MR and 75.9% had less than moderate MR	NR
Tang et al. [52]	2021	Observational	38,166 patients with CS, 596 matched patients in MitraClip and non-MitraClip arms	Patients with CS receiving MitraClip versus standard of care	In hospital: significantly lower mortality in the MitraClip cohort (OR: 0.60; 95%CI: 0.47–0.77; *p* < 0.001) 1-year: significantly lower mortality in the MitraClip cohort (HR: 0.76; 95%CI; 0.65–0.88; *p* < 0.001)	NR	NR
Haberman et al. [53]	2024	Observational	23 patients undergoing urgent TEER	100% AMI with PMR, 87% had CS	At discharge, 70% of patients were alive	Post-procedurally, MR grade 0 or 1+ was achieved in 57% of patients and MR grade to 2+ 30% of patients	At discharge, 47.8% were NYHA class I/II
Perel et al. [54]	2023	Observational	49 patients with AMI and PMR	17 patients (35%) underwent urgent TEER; 32 elective TEER	6-months: urgent TEER: 5.9%, elective TEER: 0%, non-significant difference	Post-procedurally, 93.8% of patients had MR grade ≤ 2+	Post-procedurally, 78.5% of patients were NYHA class I/II

Abbreviations: MR: mitral regurgitation; TEER: transcatheter edge-to-edge repair; CV: cardiovascular; AMI: acute myocardial infarction; NYHA: New York Heart Association; CS: cardiogenic shock; ACS: acute coronary syndrome; MCS: mechanical circulatory support; PMR: papillary muscle rupture; OR: odds ratio; HR: hazard ratio; CI: confidence interval; NR: not reported.

## Data Availability

As this is a review article, no new data were created.
